# Correlates of the discrepancy between objective and subjective cognitive functioning in non-demented patients with Parkinson’s disease

**DOI:** 10.1007/s00415-021-10519-4

**Published:** 2021-03-15

**Authors:** Mattia Siciliano, Lugi Trojano, Rosa De Micco, Valeria Sant’Elia, Alfonso Giordano, Antonio Russo, Luca Passamonti, Gioacchino Tedeschi, Carlo Chiorri, Alessandro Tessitore

**Affiliations:** 1 Department of Advanced Medical and Surgical Sciences, MRI Research Center Vanvitelli-FISM, University of Campania “Luigi Vanvitelli”, Piazza Miraglia 2, 80138 Naples, Italy; 2 Department of Psychology, University of Campania “Luigi Vanvitelli”, Viale Ellittico 31, 81100 Caserta, Italy; 3 Department of Clinical Neurosciences, University of Cambridge, CB2 0SZ Cambridge, UK; 4 Institute of Molecular Bioimaging and Physiology, CNR, Milan, Italy; 5 Department of Educational Sciences, University of Genova, Genova, Italy

**Keywords:** Fatigue, Depression, Cognitive impairment, Subjective cognitive decline, Mild cognitive impairment

## Abstract

**Background:**

Subjective complaints of cognitive deficits are not necessarily consistent with objective evidence of cognitive impairment in Parkinson’s disease (PD). Here we examined the factors associated with the objective-subjective cognitive discrepancy.

**Methods:**

We consecutively enrolled 90 non-demented patients with PD who completed the Parkinson’s Disease Cognitive Functional Rating Scale (subjective cognitive measure) and the Montreal Cognitive Assessment (MoCA; objective cognitive measure). The patients were classified as “Overestimators”, “Accurate estimators”, and “Underestimators” on the basis of the discrepancy between the objective vs. subjective cognitive measures. To identify the factors distinguishing these groups from each other, we used chi-square tests or one-way analyses of variance, completed by logistic and linear regression analyses.

**Results:**

Forty-nine patients (54.45%) were classified as “Accurate estimators”, 29 (32.22%) as “Underestimators”, and 12 (13.33%) as “Overestimators”. Relative to the other groups, the “Underestimators” scored higher on the Fatigue Severity Scale (FSS), Beck Depression Inventory (BDI), and Parkinson Anxiety Scale (*p* < 0.01). Logistic regression confirmed that FSS and BDI scores distinguished the “Underestimators” group from the others (*p* < 0.05). Linear regression analyses also indicated that FSS and BDI scores positively related to objective-subjective cognitive discrepancy (*p* < 0.01). “Overestimators” scored lower than other groups on the MoCA’s total score and attention and working memory subscores (*p* < 0.01).

**Conclusion:**

In more than 45% of consecutive non-demented patients with PD, we found a ‘mismatch’ between objective and subjective measures of cognitive functioning. Such discrepancy, which was related to the presence of fatigue and depressive symptoms and frontal executive impairments, should be carefully evaluated in clinical setting.

**Supplementary Information:**

The online version contains supplementary material available at 10.1007/s00415-021-10519-4.

## Introduction

Cognitive impairment is a frequent, pervasive and progressive non-motor manifestation of Parkinson’s disease (PD) [1]. Considering the negative impact of cognitive deficits on patients’ quality of life and functional independence [2], the International Parkinson and Movement Disorders Society (MDS) proposed consensus criteria for identifying mild cognitive impairment (MCI) in PD. This syndrome is characterized by objective cognitive deficits in addition to subjective complaints of cognitive impairments observed by either the patient, informant or clinician [1] and occurs in approximately 40% of PD population [3].

However, it is difficult to rely on patients’ subjective reports of cognitive functioning, as around half of the patients with PD tend to judge their own cognitive performances as better or worse than their actual performances on objective cognitive testing [4].

Previous studies on the relationships between subjective cognitive complaints and objective cognitive impairment in PD led to conflicting results. Some authors did not observe statistically significant associations between subjective and objective cognitive functioning (e.g., Refs. [5, 6]). Others showed that subjective cognitive complaints tended to be more frequent in patients who later developed MCI or dementia (33–70%), although the percentage of patients who complained about cognitive deficits but did not develop MCI or dementia was also high [7–14].

The opposite situation, i.e., a lack of subjective cognitive complaints in patients with objective cognitive impairments [4], has also been reported in PD. This ‘cognitive anosognosia’ is linked to disease progression and is tightly related to deficits in frontal lobe and executive functioning [4, 15].

As self-awareness of cognitive performance is also associated to motivation and emotional processing [16], the objective-subjective cognitive discrepancy in PD has been hypothesized to be moderated by psychiatric and behavioral symptoms [17].

To date, compelling evidence supports the idea that depression contributes to the objective-subjective cognitive discrepancy [4, 9, 12, 14, 15, 18–20]. However, no conclusive evidence is available regarding the possible relationship with other common behavioral symptoms in PD (e.g., apathy, anxiety, fatigue, sleep disorders) or demographic and clinical features. For example, some studies showed that subjective cognitive complaints were more strongly related to anxiety [21] or fatigue [22] than to objective cognitive impairment, although other studies did not confirm this relationship [19]. These conflicting results may have stemmed from differences in the experimental designs and methodological approaches across studies, for instance the use of brief vs. more comprehensive behavioral evaluations. Moreover, only a few studies [14, 19] considered the potential impact of sleep disorders on the objective-subjective cognitive discrepancy. Yet evidence in healthy elderly [23] suggested that subjective cognitive measures were unlikely to provide accurate estimates of objective cognitive functioning in presence of sleep disturbances.

Therefore, a comprehensive evaluation of behavioral symptoms as well as of demographic and clinical features is necessary to identify the factors correlated with objective-subjective cognitive discrepancy. Thus far, only two studies have used this approach and both reported a close relationship between depression and objective-subjective cognitive discrepancy. However, one study used a small sample size (*n* = 70; [19]), while the other employed an informant-based behavioral scale [4, 24], that is known to potentially underestimate symptom severity [25].

To clarify the factors related to the objective-subjective cognitive discrepancy has implications for clinical practice and planning therapeutic strategies in PD. In clinical settings, where time resources are typically restricted, it is crucial to decide which depth of neuropsychological assessment is needed (i.e., level I screening vs. level II comprehensive evaluation; [1]). This clinical decision-making can be guided by the knowledge about the most important factors in determining cognitive deficits in PD, including the potential discrepancy between objective vs. subjective cognitive functioning.

In this study, we explored the main demographic, clinical, and patient-reported behavioral factors that were possibly associated to the objective-subjective cognitive discrepancy in a cohort of consecutive non-demented patients with PD.

Based on previous studies [9, 18, 26], we expected that mood disturbances severity could increase the discrepancy between objective and subjective cognitive functioning.

## Methods

### Patients and procedures

One hundred eligible patients with a clinical diagnosis of idiopathic PD were consecutively screened at the Movement Disorders outpatient clinic of the First Division of Neurology, University of Campania “Luigi Vanvitelli” (Naples, Italy).

Exclusion criteria were: (1) history of cerebrovascular disorder or major and unstable medical disease; (2) lifetime or current psychotic disorders including major depressive episode, ascertained via the Mini International Neuropsychiatric Inventory [27]; (3) dementia, following the level I testing procedures proposed by MDS Task Force [28], in terms of co-occurrence of decreased global cognitive efficiency (i.e., age- and education-adjusted MoCA total score below Italian cut-off of 15.5 points; [29]), impairment in more than one cognitive domain (i.e., at least two age- and education-adjusted MoCA cognitive domain scores below Italian scores; [29]), and cognitive deficiency severe enough to impair daily life activities (based on medical records of patients’ and caregivers’ reports).

The local Ethical Committee supervised and approved all the procedures, following the Declaration of Helsinki. All participants gave their written informed consent before their inclusion in the study.

### Demographics and clinical features

In all patients, we collected the following demographic characteristics: age, education, and sex. To assess the severity of motor symptoms, we used the motor section of the Unified Parkinson’s Disease Rating Scale (UPDRS; [30]) and the Hoehn and Yahr staging system (HY; [31]). The patients were assessed in the “ON” state, and their medication regimen was recorded. Daily levodopa equivalent dosage (LEDD_L-DOPA_), daily dopamine agonist equivalent dosage (LEDD_DA_), and the total amount of dopaminergic medication (LEDD total) were computed using Tomlinson et al.’s algorithm [32].

### Behavioral measures

To characterize the behavioral profile of patients with PD, we used the Fatigue Severity Scale (FSS) [33, 34], the Beck Depression Inventory (BDI; [35]), the self-rated version of Parkinson Anxiety Scale [36, 37], and the self-rated version of Apathy Evaluation Scale (AES; [38, 39]). In addition, sleep problems were assessed via the Epworth Sleepiness Scale (ESS [40];) and the Parkinson’s Disease Sleep Scale (PDSS; [41]).

### Cognitive assessment

Objective cognitive functioning was assessed with the Montreal Cognitive Assessment (MoCA; [29, 42]), which provides a total score and six subscores for selected cognitive domains. The total scores were converted in age- and education-adjusted *Z* scores (MoCA adjusted *Z* scores; [29]). Higher MoCA adjusted *Z* scores indicate better objective cognitive performance. The cut-off for the presence of objective cognitive impairment was a MoCA adjusted *Z* score ≤ − 1.5.

Subjective cognitive complaints were assessed with the patient form of the Parkinson’s Disease-Cognitive Functional Rating Scale (PD-CFRS; [43, 44]). This is a self-report measure of cognitive dysfunction assessing the degree to which cognitive symptoms interfered with instrumental daily activities over the past 2 weeks. The PD-CFRS raw scores were converted in *Z* scores [45], and then multiplied by − 1 so to obtain *Z* scores (PD-CFRS *Z* score) with higher values indicating better subjective cognitive functioning. The cut-off for the presence of clinically significant subjective cognitive complaints was a PD-CFRS *Z* score ≤ − 1.5.

The discrepancy between objective and subjective cognitive functioning was computed as the difference between the MoCA adjusted *Z* scores and the PD-CFRS *Z* scores.

On the basis of MoCA and PD-CFRS *Z* score cut-offs, patients were categorized into three groups: “Underestimators”, i.e., patients with subjective cognitive complaints but no objective cognitive impairment (i.e., PD-CFRS *Z* score ≤ − 1.5 and MoCA adjusted *Z* score ≥ − 1.5); “Accurate estimators”, i.e., patients with neither subjective cognitive complaints nor objective cognitive impairment (i.e., PD-CFRS *Z* score and MoCA adjusted *Z* score ≥ − 1.5) or, alternatively, with both subjective cognitive complaints and objective cognitive impairment (i.e., PD-CFRS *Z* score and MoCA adjusted *Z* score ≤ − 1.5); “Overestimators”, patients with objective cognitive impairment but no subjective cognitive complaints (i.e., PD-CFRS adjusted *Z* score ≥ − 1.5 and MoCA adjusted *Z* score ≤ − 1.5).

Our definition of “Underestimators” is similar to the one of “Subjective Cognitive Complaint” [14, 19] or “Subjective Cognitive Decline” [13, 46] which is often reported in the PD and Alzheimer’s disease literature. However, here, we used the term “Underestimators” in an operative sense, without diagnostic implications. Likewise, our use of the term “Overestimators” might recall the concept of “cognitive anosognosia” [4] but also in this case we used the term “Overestimators” in an operational acceptation.

### Statistical analyses

The data were tested for normality and values between − 1 and 1 for asymmetry and kurtosis were considered acceptable.

Cohen’s kappa (*κ*) was calculated as a measure of agreement between the patients’ subjective cognitive complaints (presence vs. absence) and objective evidence of cognitive impairment (presence vs. absence). The strength of agreement was interpreted as follows: *κ* < 0.00 poor; 0.00 ≤ *κ* ≤ 0.20 slight; 0.21 ≤ *κ* ≤ 0.40 fair; 0.41 ≤ *κ* ≤ 0.60 moderate; 0.61 ≤ *κ* ≤ 0.80 substantial; 0.81 ≤ *κ* ≤ 1.00 almost perfect [47].

We compared the groups of “Accurate estimators”, “Underestimators”, and “Overestimators” in terms of demographic, clinical, and behavioral features using Pearson’s chi-square tests (χ2) for categorical variables and one-way analyses of variance (ANOVA) for continuous variables. Pairwise post-hoc comparisons were used to determine which groups were significantly different and the directionality of the effects.

To identify the demographic, clinical, and behavioural features of the group of “Underestimators”, we first carried out simple binary logistic regression analyses, to identify which features were able to discriminate the group of “Underestimators” from that of the “Non-underestimators” (i.e., “Overestimators” and “Accurate estimators” grouped together as in Ref. [48]), at a bivariate level. Second, we entered the features that showed a significant bivariate association with group membership in a multiple binary logistic regression analysis model (forced entry method) to test which ones independently contributed to explaining patient classification.

The associations between the demographic, clinical, and behavioral features and the objective-subjective cognitive discrepancy (difference between the MoCA *Z* scores and PD-CFRS) was investigated using bivariate and multiple linear regression analyses (forced entry method).

To check the reliability of our findings, a bootstrap approach (1000 bootstrap) with a 95% bias corrected and accelerated confidence intervals [95% CI] was used. The bias of an estimate can be ignored if it is lower than 0.25 times its standard error [49].

Since the subjective cognitive complaints might have different implications in patients with or without objective cognitive impairment, we repeated all the analyses after excluding from the “Accurate estimators” group the patients with both subjective cognitive complaints and objective cognitive impairment.

All multiple comparisons were corrected for familywise errors by Bonferroni’s procedures; Bonferroni corrected *p *value < 0.05 were considered statistically significant.

All statistics were performed using Statistical Package for Social Science version 20 (SPSS, Chicago, IL), All figures were created by GraphPad Prism 6.0 and Matlab.

## Results

Out of 100 screened patients, eight were not willing to participate in our study, whereas two patients were excluded (of these one suffered from a current major depressive episode, and the other was diagnosed with PD-dementia).

The demographic and clinical features of included patients (*n* = 90) did not differ from those of screened patients who were not included (*n* = 10) (Supplementary Table 1).

The descriptive statistics of our final sample (*n* = 90) were reported in Table 1.

**Table 1:** Overall sample descriptive statistics (*n* = 90)

Variable	Mean (SD) or count (%)
Demographics	
Age	66.74 (9.22)
Education, years	9.66 (4.09)
Sex, male	53 (58.90%)
Clinical features	
Age at onset	61.63 (9.72)
Disease duration, years	5.25 (2.92)
UPDRS-III	27.33 (9.57)
Hoehn and Yahr stage	2.00 (0.37)
LEDD total (mg/day)	516.82 (233.37)
LEDD_DA_ (mg/day)	71.23 (106.27)
LEDD_L-DOPA_ (mg/day)	386.16 (259.29)
Behavioural measures	
Fatigue Severity Scale	3.47 (1.88)
Beck Depression Inventory	8.91 (7.45)
Parkinson Anxiety Scale	11.92 (9.26)
Apathy Evaluation Scale	31.74 (7.55)
Parkinson’s disease sleep scale	114.95 (22.23)
Epworth Sleepiness Scale	5.77 (4.19)
Cognitive assessment	
MoCA total	
Raw score	20.02 (4.87)
Adjusted score^a^	22.46 (4.31)
Adjusted *Z* score^a^	0.18 (1.42)
MoCA adjusted subscores^a^	
Memory	1.29 (1.47)
Visuospatial abilities	0.68 (1.40)
Executive functions	0.78 (1.77)
Attention, and WM	5.01 (1.03)
Language	3.22 (1.52)
Orientation	5.76 (0.57)
PD-CFRS	
Raw	2.42 (3.01)
*Z* score	− 0.68 (2.32)
MoCA (*Z* score) minus PD-CFRS (*Z* score)	− 0.64 (2.30)

*SD* standard deviation, *UPDRS* Unified Parkinson’s Disease Rating Scale, *LEDD* levodopa equivalent daily dose, *MoCA* Montreal Cognitive Assessment, *WM* working memory, *PD-CFRS* Parkinson’s Disease Cognitive Functional Rating Scale

^a^Adjusted according to age, education, or sex

Asymmetry and kurtosis were acceptable for all continuous variables.

There was poor agreement between the patients’ subjective cognitive complaints and objective evidence of cognitive impairment (*κ* = − 0.23, *p* = 0.01).

**Fig. 1:**
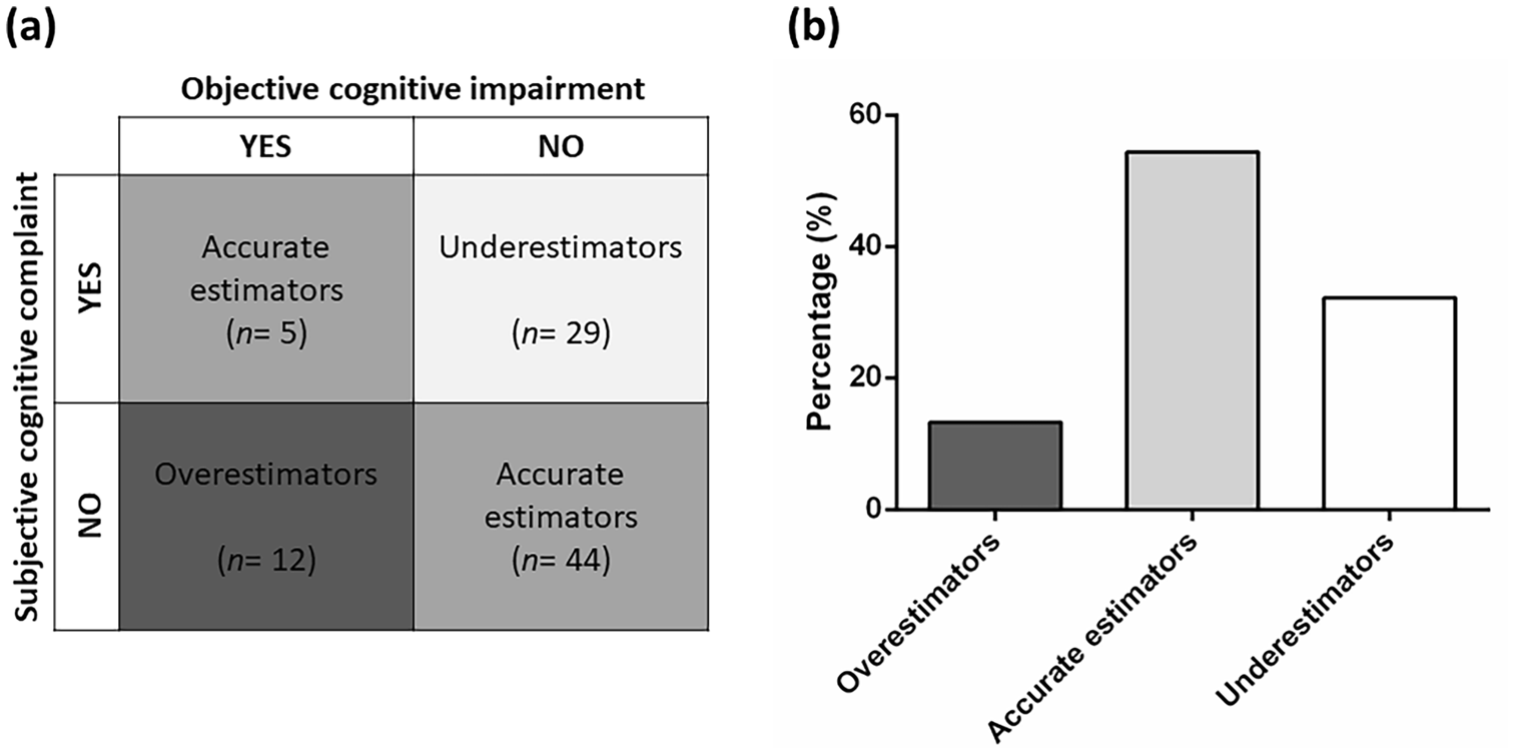
**a** Patients grouped according to the presence of objective cognitive impairment and subjective cognitive complaint. **b** Percentage of overestimators, accurate estimators, and underestimators

Of the final sample, 49 patients (54.45%) were classified as “Accurate estimators”, 29 (32.22%) as “Underestimators”, and 12 (13.33%) as “Overestimators” (Fig. 1a, b).

One-way ANOVAs and χ2 test, complemented by post-hoc analyses, did not show statistically significant differences among “Overestimators”, “Accurate estimators”, and “Underestimators” in terms of demographic and clinical features. The group of “Underestimators” scored higher on the FSS, BDI, and PAS relative to the group of “Overestimators” and “Accurate estimators”. The latter two groups did not differ between each other in terms of any behavioral feature considered. The group of “Underestimators” scored lower on the PD-CFRS than the group of “Overestimators” and “Accurate estimators”. Relatively to the other groups, the group of “Overestimators” scored lower on the MoCA total score and the attention and working memory subscore of the MoCA (Table 2).

**Table 2:** Comparisons among groups on demographics, clinical, and behavioural features

Variable	Overestimators (*n* = 12)	Accurate estimators (*n* = 49)	Underestimators (*n* = 29)	*F*/χ^2^	*p* value	Adj-*p*^c^	*η*^2^ or *ϕc*	Bonferroni-adjusted post-hoc analyses		
	Mean (SD) or *n* (%)	Mean (SD) or *n* (%)	Mean (SD) or *n* (%)					O vs. A (*p* value)	O vs. U (*p* value)	A vs. U (*p* value)
Demographics										
Age	63.92 (9.38)	66.73 (9.16)	67.93 (9.31)	0.80	0.452	1.000	0.01	–	–	–
Education, years	11.08 (5.36)	9.92 (3.92)	8.62 (3.67)	1.78	0.174	1.000	0.03	–	–	–
Sex, male^a^	7 (58.30%)	31 (63.30%)	15 (51.70%)	1.00	0.605	1.000	0.10	–	–	–
Clinical features										
Age at onset	57.67 (11.17)	61.84 (9.49)	62.96 (9.39)	1.28	0.283	1.000	0.02	–	–	–
Disease duration, years	6.25 (4.75)	4.90 (2.29)	5.43 (2.93)	1.11	0.334	1.000	0.02	–	–	–
UPDRS-III	27.17 (9.21)	26.82 (10.21)	28.29 (8.77)	0.20	0.813	1.000	0.00	–	–	–
Hoehn and Yahr stage	2.08 (0.51)	1.94 (0.37)	2.07 (0.26)	1.52	0.224	1.000	0.03	–	–	–
LEDD total (mg/day)	628.92 (263.45)	475.22 (215.51)	541.57 (239.35)	2.39	0.097	1.000	0.05	–	–	–
LEDD_DA_ (mg/day)	56.00 (72.45)	86.80 (125.36)	49.74 (74.22)	1.20	0.304	1.000	0.02	–	–	–
LEDD_L-DOPA_ (mg/day)	535.42 (274.78)	333.16 (237.13)	416.00 (269.65)	3.36	0.039	0.942	0.07	–	–	–
Behavioural measures										
Fatigue Severity Scale	3.05 (2.15)	2.87 (1.68)	4.65 (1.55)	10.25	< 0.001	**<** **0.001**	0.19	1.000	**0.024**	**<** **0.001**
Beck Depression Inventory	6.23 (7.03)	6.65 (6.53)	13.83 (6.85)	11.54	< 0.001	**<** **0.001**	0.21	1.000	**0.004**	**<** **0.001**
Parkinson Anxiety Scale	10.17 (9.01)	9.14 (7.82)	17.34 (9.52)	8.65	< 0.001	**0.008**	0.16	1.000	**0.049**	**<** **0.001**
Apathy Evaluation Scale	28.90 (9.36)	32.00 (7.34)	32.28 (7.30)	0.80	0.451	1.000	0.01	–	–	–
Parkinson’s disease sleep scale	114.91 (18.67)	117.48 (23.71)	110.33 (20.93)	0.79	0.454	1.000	0.02	–	–	–
Epworth Sleepiness Scale	7.29 (5.86)	5.03 (3.65)	6.40 (4.14)	1.92	0.152	1.000	0.04	–	–	–
Cognitive assessment										
MoCA total score^b^	16.43 (5.10)	23.12 (3.23)	23.84 (3.56)	19.46	< 0.001	**<** **0.001**	0.30	**<** **0.001**	**<** **0.001**	1.000
MoCA subscores^b^										
Memory	0.58 (0.99)	1.55 (1.60)	1.14 (1.30)	2.38	0.098	1.000	0.05	–	–	–
Visuospatial abilities	0.49 (1.25)	0.79 (1.37)	0.56 (1.51)	0.35	0.699	1.000	0.00	–	–	–
Executive functions	0.47 (2.22)	0.86 (1.78)	0.75 (1.61)	0.23	0.793	1.000	0.00	–	–	–
Attention, and WM	4.03 (1.55)	5.13 (0.85)	5.22 (0.85)	7.19	0.001	**0.030**	0.14	**0.002**	**0.002**	1.000
Language	2.99 (1.64)	3.29 (1.57)	3.20 (1.42)	0.18	0.833	1.000	0.00	–	–	–
Orientation	5.36 (0.74)	5.88 (0.41)	5.72 (0.65)	4.48	0.014	0.335	0.09	–	–	–
PD-CFRS score	0.83 (0.93)	1.24 (1.86)	5.07 (3.44)	25.77	< 0.001	**<** **0.001**	0.37	1.000	**<** **0.001**	**<** **0.001**

Statistically significant differences are shown in bold

*ϕc* Cramér’s *V*, *η*^*2*^ partial eta squared, *SD* standard deviation, *UPDRS* Unified Parkinson’s Disease Rating Scale, *LEDD* levodopa equivalent daily dose, *O* overestimators, *A* accurate estimators, *U* underestimators, *MoCA* Montreal Cognitive Assessment, *PD-CFRS* Parkinson’s Disease Cognitive Functional Rating Scale

^a^Categorical variable

^b^Adjusted according to age, education, or sex

^c^Adj-*p* represents *p* value corrected for multiple comparisons using the Bonferroni procedure

The simple binary logistic regression analyses showed that neither the demographic nor the clinical features could distinguish the group of “Underestimators” from that of the “Non-underestimators” (i.e., “Overestimators” or “Accurate estimators” grouped together as in Ref [48]). In contrast, the FSS, BDI, PAS, and AES were able to distinguish the “Underestimators” from “Non-underestimators”. However, the multiple binary logistic regression analysis revealed that only the BDI and FSS were independently able to classify the “Underestimators” vs. “Non-underestimators” with an overall accuracy of 76% (Table 3).

**Table 3:** Simple and multiple binary logistic regression analyses assessing which demographics, clinical, and behavioural features distinguished underestimators from non-underestimators; 95% bias corrected and accelerated confidence intervals [95% CI] (1000 bootstrap samples) for the logistic regression coefficients were reported in parentheses

Variable	Estimate [CI 95%]	Bias	SE	*p* value^b^	OR [CI 95%]
Simple regression					
Demographics					
Age	0.02 [− 0.03, 0.07]	0.00	0.02	0.399	1.02 [0.97, 1.07]
Education, years	− 0.09 [− 0.22, 0.01]	− 0.00	0.06	0.101	0.90 [0.80, 1.01]
Sex^a^	− 0.43 [− 1.29, 0.50]	0.04	0.45	0.342	0.64 [0.26, 1.58]
Clinical features					
Age at onset	0.02 [− 0.02, 0.07]	− 0.00	0.02	0.379	1.02 [0.97, 1.07]
Disease duration, years	0.03 [− 0.13, 0.20]	− 0.00	0.08	0.691	1.03 [0.88, 1.19]
UPDRS-III	0.01 [− 0.02, 0.06]	0.00	0.02	0.520	1.01 [0.96, 1.06]
Hoehn and Yahr stage	0.80 [− 0.27, 2.49]	0.40	2.74	0.222	2.22 [0.61, 8.07]
LEDD total (mg/day)	0.00 [− 0.00, 0.00]	0.00	0.00	0.496	1.00 [0.99, 1.00]
LEDD_DA_ (mg/day)	− 0.00 [− 0.00, 0.00]	0.00	0.00	0.212	0.99 [0.99, 1.00]
LEDD_L-DOPA_ (mg/day)	0.00 [− 0.00, 0.00]	0.00	0.00	0.471	1.00 [0.99, 1.00]
Behavioural measures					
Fatigue Severity Scale	0.57 [0.30, 0.93]	0.02	0.15	**<** **0.001**	1.77 [1.32, 2.38]
Beck Depression Inventory	0.15 [0.08, 0.26]	0.01	0.05	**<** **0.001**	1.15 [1.07, 1.24]
Parkinson Anxiety Scale	0.10 [0.05, 0.16]	0.00	0.02	**<** **0.001**	1.10 [1.04, 1.16]
Apathy Evaluation Scale	0.10 [0.03, 0.19]	0.00	0.04	**0.024**	1.10 [1.01, 1.20]
Parkinson’s disease sleep scale	− 0.01 [− 0.04, 0.00]	− 0.00	0.01	0.228	0.98 [0.96, 1.00]
Epworth Sleepiness Scale	0.05 [− 0.05, 0.18]	0.00	0.06	0.327	1.05 [0.94, 1.16]
Multiple regression^c^					
Fatigue Severity Scale	0.57 [0.09, 1.44]	0.05	0.35	**0.008**	1.77 [1.16, 2.71]
Beck Depression Inventory	0.11 [0.01, 0.32]	0.01	0.07	**0.035**	1.12 [1.00, 1.25]
Parkinson Anxiety Scale	0.01 [− 0.08, 0.10]	0.00	0.05	0.702	1.01 [0.93, 1.09]
Apathy Evaluation Scale	− 0.06 [− 0.20, 0.04]	− 0.01	0.06	0.938	0.95 [0.85, 1.02]

Statistically significant variables are shown in bold

*SE* standard error, *OR* odds ratio, *CI* confidence interval, *UPDRS* Unified Parkinson’s Disease Rating Scale, *LEDD* levodopa equivalent daily dose

^a^Coded as: 0 = male, 1 = female

^b^*p* value related to unstandardized beta coefficient using the Wald statistic

^c^Model χ^2^ (4) = 33.01, *p* value < 0.01, *R*^2^ = 0.44 (Nagelkerke)

**Fig. 2:**
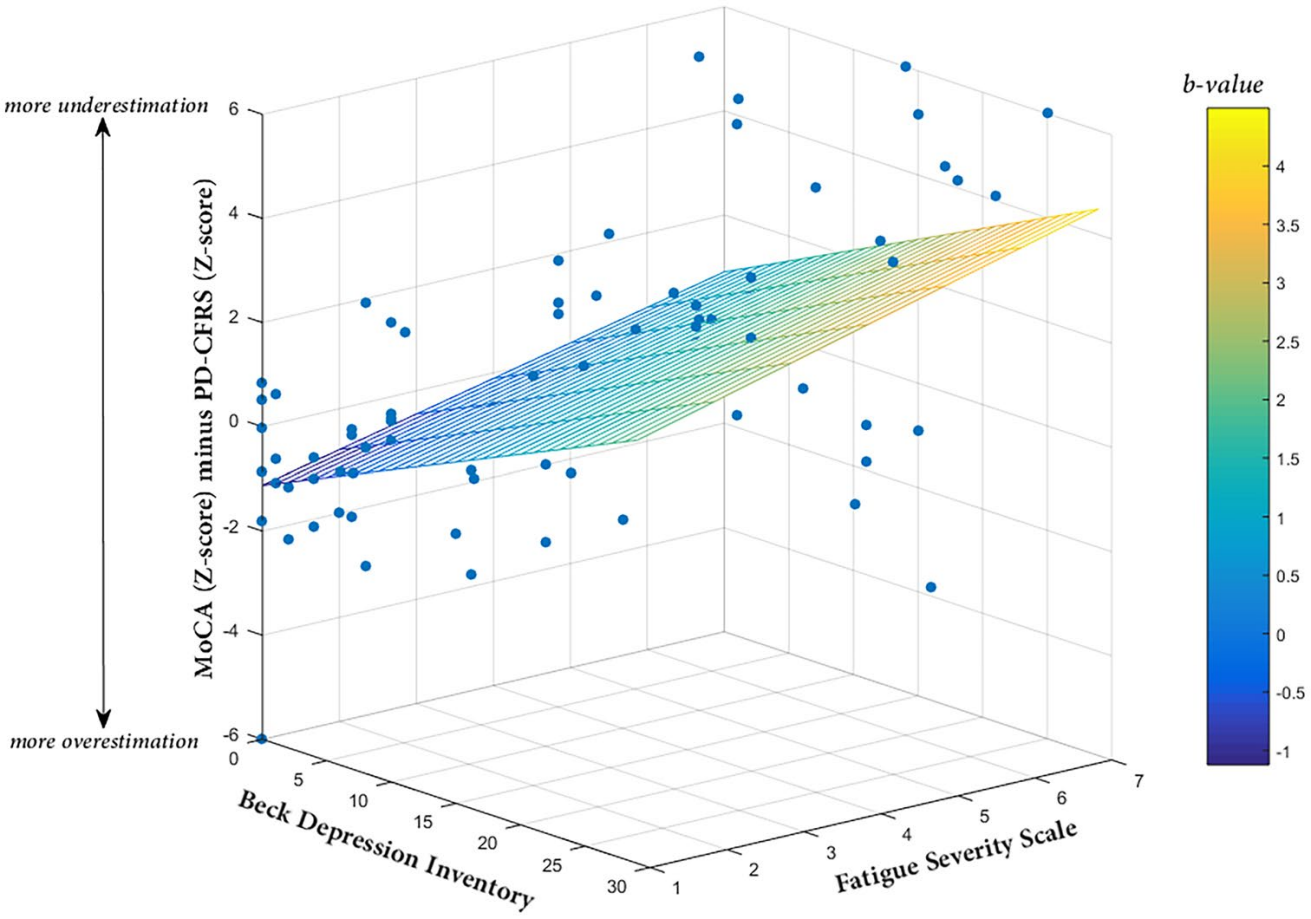
A 3-D scatterplot showing the regression of MoCA (*Z* score) minus PD-CFRS (*Z* score) on Beck Depression Inventory and Fatigue Severity Scale; the tinted trapezium (regression plane) is built by linear multiple regression equation and the dots represent the observed data points; the color bar represents the values of beta coefficients

The bivariate linear regression analyses showed that higher scores on FSS, BDI, PAS, AES, and ESS were associated with a greater discrepancy between PD-CFRS *Z* scores and the MoCA adjusted *Z* scores. The multiple linear regression analysis indicated that only the FSS and BDI were significantly associated with a discrepancy between the MoCA *Z* scores and the PD-CFRS *Z* scores and with an overall model accuracy of 42% (Table 4; Fig. 2).

**Table 4:** Simple and multiple linear regression analyses assessing which demographics, clinical, and behavioural features were associated with the discrepancy between objective and subjective cognitive functioning (MoCA *Z* scores minus PD-CFRS *Z* scores) in overall sample; 95% bias corrected and accelerated confidence intervals [95% CI] (1000 bootstrap samples) for the linear regression coefficients were reported in parentheses

Variable	Estimate [CI 95%]	SE	Bias	*β*	*p* value
Simple regression					
Demographics					
Age	0.02 [− 0.03, 0.07]	0.02	0.00	0.07	0.468
Education, years	− 0.09 [− 0.18, 0.04]	0.05	0.00	− 0.14	0.186
Sex^a^	− 0.23 [− 1.26, 0.74]	0.52	− 0.00	− 0.04	0.674
Clinical features					
Age at onset	0.03 [− 0.02, 0.09]	0.02	0.00	0.12	0.236
Disease duration, years	− 0.15 [− 0.32, 0.08]	0.10	0.01	− 0.17	0.096
UPDRS-III	0.00 [− 0.04, 0.04]	0.02	0.00	− 0.02	0.799
Hoehn and Yahr stage	0.26 [− 0.80, 1.33]	0.53	− 0.02	0.03	0.721
LEDD total (mg/day)	− 0.00 [− 0.00, 0.00]	0.00	0.00	− 0.07	0.505
LEDD_DA_ (mg/day)	− 0.00 [− 0.00, 0.00]	0.00	0.00	− 0.08	0.422
LEDD_L-DOPA_ (mg/day)	0.00 [− 0.00, 0.00]	0.00	0.00	− 0.04	0.714
Behavioural measures					
Fatigue Severity Scale	0.69 [0.39, 0.99]	0.15	− 0.00	0.51	**<** **0.001**
Beck Depression Inventory	0.18 [0.10, 0.25]	0.03	0.00	0.53	**<** **0.001**
Parkinson Anxiety Scale	0.11 [0.05, 0.16]	0.02	0.00	0.40	**<** **0.001**
Apathy Evaluation Scale	0.09 [0.03, 0.16]	0.03	0.00	0.27	**0.010**
Parkinson’s disease sleep scale	− 0.01 [− 0.04, 0.00]	0.01	0.00	− 0.15	0.171
Epworth Sleepiness Scale	0.15 [0.01, 0.34]	0.08	0.00	0.25	**0.015**
Multiple regression^b^					
Fatigue Severity Scale	0.42 [0.09, 0.82]	0.17	0.00	0.31	**0.007**
Beck Depression Inventory	0.13 [0.03, 0.23]	0.05	0.00	0.39	**0.004**
Parkinson Anxiety Scale	0.00 [− 0.05, 0.06]	0.02	− 0.00	0.02	0.823
Apathy Evaluation Scale	− 0.01 [− 0.07, 0.04]	0.03	0.00	− 0.05	0.575
Epworth Sleepiness Scale	0.00 [− 0.10, 0.11]	0.07	0.00	0.00	0.929

Statistically significant variables are shown in bold

^a^Coded as: 0 = male, 1 = female

^b^Model (*F* test) = 10.89, *p* value < 0.001, *R*^2^ = 0.42

*SE* standard error, *CI* confidence interval, *UPDRS* Unified Parkinson’s Disease Rating Scale, *LEDD* levodopa equivalent daily dose

The results were confirmed after the exclusion of patients who showed a congruency between subjective cognitive complaints and objective cognitive impairment (*n* = 5) from the “Accurate estimators” group (Supplementary Tables 2, 3, 4, 5).

The bootstrapping procedure did not reveal substantial biases, and confirmed that our sample size was adequate to detect statistically meaningful effects [49].

## Discussion

We studied the extent to which the objective-subjective cognitive discrepancy in PD was associated with demographic, clinical, and patient-reported behavioral features.

Three main results emerged. First, there was poor agreement between patient subjective reports and patient objective deficits, as > 45% of patients showed objective-subjective cognitive discrepancy. Second, the underestimation of cognitive performance in PD was associated with the severity of fatigue and depressive symptoms but not with the demographics or clinical features. This supports the idea that specific behavioral symptoms are the main correlates of objective-subjective cognitive discrepancy in PD [4, 14, 19, 21]. Third, the overestimation of cognitive performance was tightly associated with frontal executive impairments.

Our first main finding demonstrated that the objective-subjective cognitive discrepancy is a remarkable clinical phenomenon in PD. Indeed, 32% of patients had subjective cognitive complaints but no objective cognitive impairment (“Underestimators”), while a 13% showed an inverse pattern (i.e., objective cognitive impairment but no subjective cognitive complaint, “Overestimators”). These findings highlight that underestimation of one’s own cognitive abilities is common and clinically relevant in PD [13, 14], consistent with a recent study, in which the objective-subjective cognitive discrepancy occurred in 45% of patients with PD, with a higher percentage (24%) of patients underestimating their objective cognitive abilities and a lower percentage (21%) overestimating it [4].

These results showed that not all PD patients with objective cognitive impairments are able to report their real cognitive performance, whereas not all PD patients reporting cognitive problems display ‘objective’ deficits [5]. As the clinical diagnosis of MCI in PD sometimes relies on cognitive impairments typically reported by the patient or informant [1], our findings suggest caution in relying on patients’ subjective reports, especially in the absence of objective testing [6].

Our second main finding showed that severity of fatigue, depressive, and anxious symptoms distinguished “Underestimators” from “Overestimators” and “Accurate estimators”. The link between cognitive underestimation and fatigue has been consistently demonstrated in patients with multiple sclerosis (e.g., Refs. [48, 50]). However, this association has been scarcely investigated in PD, and conflicting results have been reported [19, 22]. In PD, Kluger et al. [51] proposed that fatigue, defined as a significantly diminished level of energy or increased perception of effort that is disproportionate to attempted activities [52], may be linked more to altered subjective awareness than to actual performance limitations. Indeed, fatigued patients often report subjective complaints of increased sense of effort or decreased stamina that are not related to objective decrement in performance [51]. In keeping with these findings, our group of “Underestimators” reported subjective complaints of cognitive deficits which were not supported by objective evidence of cognitive impairment.

Such link between fatigue and cognitive underestimation may stem, at least in part, from altered self-awareness (e.g., exaggeration of deficits or hyperawareness). According to Rosen’s model [16], accurate self-awareness of performance depends on active monitoring of task performance, which results from comparing current performance with task demands and with the level of performance that is considered acceptable (e.g., a certain number of errors could be considered acceptable on a given task). Therefore, fatigue symptoms in “Underestimators” might be ascribed to alterations in self-awareness caused by inefficient monitoring of sensorimotor and cognitive processes. Recent evidence seems to support this speculation [53], but future studies are required.

“Underestimators” also showed more depressive and anxious symptoms than the two other groups, which is consistent with previous studies in PD [9, 12, 14, 18–20]. Together, these findings demonstrate a tight association between self-awareness of cognitive performance and emotional processing [16]. Nevertheless, it remains unclear if mood symptoms alter self-awareness or whether a pre-existing deficit in self-awareness is antecedent to the development of mood symptoms [15]. It is also possible that more severe mood symptoms reported by the “Understimators” foster a negative bias in reporting problems or contribute to exaggerating the cognitive deficits (hyperawareness). This is because a negative and pessimistic vision of oneself, the environment, and the future is at the core of several mood disturbances [54].

Our third main finding revealed that, compared to the other groups, the “Overestimators” scored lower on the attention and working memory subscore of the MoCA (deriving from cognitive subtests such as Digit Span Backward [55], Serial 7 subtractions [56]). These results reinforce the evidence of an association between frontal executive impairments and poor self-awareness of cognitive deficits in PD [4, 15, 57], which is also in line with models of anosognosia in Alzheimer’s disease [58].

Additional analyses corroborated our findings and confirmed their statistical robustness. First, we found that our main findings held when the “Overestimators” and “Accurate estimators” were merged in the same group (“Non-underestimators”) and compared to the group of “Underestimators” in logistic regression analyses. Second, we obtained similar results when we explored the factors associated with objective-subjective cognitive discrepancy (i.e., the difference between the MoCA *Z* scores and PD-CFRS) via the linear regression analyses in the whole patient sample.

When all the behavioural symptoms were evaluated simultaneously (by logistic or linear multiple regression analyses), depressive but not anxious symptoms were associated with the objective-subjective cognitive discrepancy. These results suggested that specific facets of depression, rather than those shared with anxiety (e.g., loss of energy) [59], may play a pivotal role in objective-subjective cognitive discrepancy. Interestingly, patients who are depressed, but not necessarily anxious, typically express negative beliefs about themselves (e.g., cognitive underestimation), the world, and the future [60].

All these findings highlight the importance of assessing and monitoring fatigue and depressive symptoms in PD, especially when patients’ complaints of cognitive impairment are used as prognostic indicators of future objective cognitive deterioration (e.g., Refs. [10, 13, 14]).

We are aware that one limitation of the present study relates to the tools used to assess subjective cognitive complaints and objective cognitive impairment. As Jessen et al. [46] highlighted, a neuropsychological battery covering all domains is necessary for accurately evaluating the ‘mismatch’ between subjective and objective cognitive performance. The PD-CFRS has been validated as a measure of self-perceived impact of cognitive changes on daily functioning [43, 45], and provides only an indirect measure of subjective complaints of cognitive impairment. PD-CFRS is available in Italian and has been used in similar studies in PD [26], although it may be less specific than other scales (for a review, see Ref. [17]). The MoCA is a cognitive screening tool but is less sensitive to domain-specific dysfunctions than comprehensive neuropsychological batteries [1]. The use of MoCA and PD-CFRS may have partially inflated the objective-subjective cognitive discrepancy. Indeed, the cognitive functions implied in daily activities, as explored by PD-CFRS, may not necessarily parallel those needed to perform a cognitive screening test. Consequently, patients classified as “Understimators” may be affected by an objective cognitive impairment undetected by MoCA. On the other hand, as MoCA is a cognitive screening test recommended by the MDS, widely used in clinical practice and research, this could help ensuring a reasonable generalization of our results [11, 20]. Moreover, we considered MoCA subscores to increase the explanatory power of our results and to provide a more nuanced assessment of objective cognitive functioning [61].

An additional limitation of this study is its cross-sectional nature which calls for replication by longitudinal study designs, by which it is possible to explore the predictive relationship between the self-experienced worsening of cognitive capacities and the objective cognitive deterioration [46]. Furthermore, to screen the presence of PD dementia, we used the level I testing procedures which, compared to level II ones, do not allow to specify the pattern and severity of the cognitive impairment and may lead to suboptimal recruitment decisions [28]. Finally, the low percentage of “Overestimators” did not enable us to use more powerful statistical methods, such as multinomial logistic regression analyses, to characterize the profile of this group.

Despite these shortcomings, our findings provide further insights on the main factors correlated with the discrepancy between objective and subjective cognitive functioning in PD. Accurate detection of cognitive impairment is crucial for guiding treatments and neuropsychological assessment in a patient-centered manner. This calls for a need of increased awareness of ‘core’ behavioral symptoms such as fatigue and depression, which are likely antecedents of the discrepancy between objective cognitive impairments and subjective cognitive complaints in PD.

The further implications of our study are threefold. First, caution should be exercised when making a clinical diagnosis of MCI, especially when MCI is diagnosed only in terms of subjectively-reported cognitive complaints [1, 6]. Second, clinicians should include an assessment of fatigue and depression in their routine cognitive examination to determine to extent to which these behavioral factors influence cognitive complains. Third, our data might suggest that behavioral and/or pharmacological interventions for fatigue and depression could reduce subjective cognitive complaints [62].

Enhancing patients’ abilities to correctly perceive their individual level of cognitive functioning has great potential to improve their own and their caregivers’ quality of life.

## Supplementary Information

Below is the link to the electronic supplementary material.

Supplementary file1 (DOCX 31 kb)

## Data Availability

Research data were not shared.
