# Supplementary material for: Correlates of the discrepancy between objective and subjective cognitive functioning in non-demented patients with Parkinson’s disease
Source: J Neurol. 2021 Mar 15;268(9):3444–55. doi: 10.1007/s00415-021-10519-4 (PMC8357714; doi:10.1007/s00415-021-10519-4)
Supplement: Supplementary file 1 — Supplementary file1 (DOCX 31 kb) [file 415_2021_10519_MOESM1_ESM.docx]

**Correlates of the discrepancy between objective and subjective cognitive functioning in Parkinson’s disease**

Mattia Siciliano^a,b^, Lugi Trojano^b^, Rosa De Micco^a^, Valeria Sant’Elia ^b^, Alfonso Giordano^a^, Antonio Russo^a^ , Luca Passamonti^c,d,*^, Gioacchino Tedeschi^a^, Carlo Chiorri^e^ Alessandro Tessitore^a,*^

^a^Department of Advanced Medical and Surgical Sciences - MRI Research Center Vanvitelli-FISM, University of Campania "Luigi Vanvitelli", Piazza Miraglia 2, 80138, Naples (IT);

^b^Department of Psychology, University of Campania "Luigi Vanvitelli", Viale Ellittico 31, 81100, Caserta (IT);

^c^Department of Clinical Neurosciences, University of Cambridge, Cambridge, UK.

^d^Institute of Molecular Bioimaging and Physiology, CNR, Milan, Italy.

^e^Department of Educational Sciences, University of Genova, Genova, Italy.

*Corresponding author:
Alessandro Tessitore MD, PhD

Luca Passamonti MD, PhD

Department of Advanced Medical and Surgical Sciences - MRI Research Center Vanvitelli-FISM, University of Campania "Luigi Vanvitelli", Piazza Miraglia 2, 80138, Naples (IT)
email: [alessandro.tessitore@unicampania.it](mailto:alessandro.tessitore@unicampania.it)

**Supplementary Table 1.** Comparison between included and excluded/refusing patients on demographic and clinical features.

| Variable | Included patients | Excluded or refusing patients | *F*-test or χ2 | *p*-value | Adj-*p* |
| --- | --- | --- | --- | --- | --- |
|  | *n*= 90 | *n*= 10 |  |  |  |
| *Demographics* |  |  |  |  |  |
| Age | 66.74 (9.22) | 64.10 (8.46) | 0.75 | 0.38 | 1.00 |
| Education, years | 9.66 (4.09) | 7.20 (2.97) | 3.37 | 0.06 | 0.69 |
| Sex, male | 37 (41.1%) | 6 (60.0%) | 1.31 | 0.25 | 1.00 |
|  |  |  |  |  |  |
| *Clinical features* |  |  |  |  |  |
| Age at onset | 61.63 (9.72) | 56.90 (7.72) | 2.20 | 0.14 | 1.00 |
| Disease duration, years | 5.25 (2.92) | 7.20 (2.74) | 4.05 | 0.04 | 0.46 |
| UPDRS-III | 27.33 (9.57) | 28.80 (10.94) | 0.20 | 0.65 | 1.00 |
| Hoehn and Yahr stage | 2.00 (0.36) | 2.15 (0.58) | 1..30 | 0.25 | 1.00 |
| LEDD total (mg/day) | 516.82 (233.36) | 655.30 (336.00) | 2.87 | 0.09 | 0.92 |
| LEDD_DA_ (mg/day) | 71.23 (106.27) | 144.00 (126.50) | 4.05 | 0.04 | 0.46 |
| LEDD_L-DOPA_ (mg/day) | 386.16 (259.29) | 451.30 (312.66) | 0.54 | 0.46 | 1.00 |

Note. data are reported as mean (standard deviation) or count (%); UPDRS, Unified Parkinson’s Disease Rating Scale; LEDD, Levodopa Equivalent Daily Dose; Adj-*p* represents *p*-value corrected for multiple comparisons using the Bonferroni procedure.

**Supplementary Table 2.** The overall sample descriptive statistics, after excluding five patients with subjective cognitive complaint and objective cognitive impairment (*n*= 85).

| Variable | Mean (SD) or Count (%) |
| --- | --- |
| *Demographics* |  |
| Age | 66.48 (9.34) |
| Education, years | 9.78 (4.09) |
| Sex, male | 48 (56.50%) |
|  |  |
| *Clinical features* |  |
| Age at onset | 61.31 (9.77) |
| Disease duration, years | 5.31 (2.96) |
| UPDRS-III | 27.49 (9.60) |
| Hoehn and Yahr stage | 2.00 (0.38) |
| LEDD total (mg/day) | 516.43 (232.25) |
| LEDD_DA_ (mg/day) | 72.90 (108.17) |
| LEDD_L-DOPA_ (mg/day) | 382.31 (259.16) |
|  |  |
| *Behavioural measures* |  |
| Fatigue Severity Scale | 3.45 (1.85) |
| Beck Depression Inventory | 8.64 (7.34) |
| Parkinson Anxiety Scale | 11.66 (9.43) |
| Apathy Evaluation Scale | 31.59 (7.40) |
| Parkinson’s disease sleep scale | 115.45 (22.21) |
| Epworth Sleepiness Scale | 5.68 (4.27) |
|  |  |
| *Cognitive assessment* |  |
| MoCA total: |  |
| Raw score | 20.45 (4.57) |
| Adjusted score^a^ | 22.73 (4.28) |
| Adjusted Z-score^a^ | 0.27 (1.41) |
| MoCA adjusted subscores:^a^ |  |
| Memory | 1.36 (1.47) |
| Visuospatial abilities | 0.75 (1.37) |
| Executive functions | 0.88 (1.74) |
| Attention, and WM | 5.07 (1.03) |
| Language | 3.36 (1.34) |
| Orientation | 5.79 (0.54) |
| PD-CFRS: |  |
| Raw | 2.25 (2.92) |
| Z-score | -0.55 (2.25) |
| MoCA (*Z*-score) minus PD-CFRS (*Z*-score) | -0.69 (2.26) |

Note. a, adjusted according to age, education, or sex; SD, Standard Deviation; UPDRS, Unified Parkinson’s Disease Rating Scale; LEDD, Levodopa Equivalent Daily Dose; MoCA, Montreal Cognitive Assessment; WM, Working Memory; PD-CFRS, Parkinson Disease Cognitive Functional Rating Scale.

**Supplementary Table 3.** Comparisons among groups on demographics, clinical, and behavioural features, after excluding five patients with subjective cognitive complaint and objective cognitive impairment

| Variable | Overestimators  (*n*= 12) | Accurate estimators  (*n*= 44) | Underestimators  (*n*= 29) |  | F/χ^2^ | *p*-value | Adj-*p* | η^2^ or *ϕc* |  | Bonferroni-adjusted post-hoc analyses | | |
| --- | --- | --- | --- | --- | --- | --- | --- | --- | --- | --- | --- | --- |
|  | Mean (SD)  or  n (%) | Mean (SD)  or  n (%) | Mean (SD)  or  n (%) |  |  |  |  |  |  | O vs A  (*p*-value) | O vs U  (*p*-value) | A vs U  (*p*-value) |
| *Demographics* |  |  |  |  |  |  |  |  |  |  |  |  |
| Age | 63.92 (9.38) | 66.23 (9.38) | 67.93 (9.31) |  | 0.81 | 0.447 | 1.000 | 0.01 |  | - | - | - |
| Education, years | 11.08 (5.36) | 10.18 (3.86) | 8.62 (3.67) |  | 2.03 | 0.137 | 1.000 | 0.05 |  | - | - | - |
| Sex, male^a^ | 7 (58.30%) | 26 (59.10%) | 15 (51.70%) |  | 0.40 | 0.816 | 1.000 | 0.07 |  | - | - | - |
|  |  |  |  |  |  |  |  |  |  |  |  |  |
| *Clinical features* |  |  |  |  |  |  |  |  |  |  |  |  |
| Age at onset | 57.67 (11.17) | 61.25 (9.56) | 62.96 (9.39) |  | 1.24 | 0.294 | 1.000 | 0.03 |  | - | - | - |
| Disease duration, years | 6.25 (4.75) | 4.98 (2.31) | 5.43 (2.93) |  | 0.90 | 0.410 | 1.000 | 0.02 |  | - | - | - |
| UPDRS-III | 27.17 (9.21) | 27.07 (10.36) | 28.29 (8.77) |  | 0.14 | 0.868 | 1.000 | 0.00 |  | - | - | - |
| Hoehn and Yahr stage | 2.08 (0.51) | 1.93 (0.39) | 2.07 (0.26) |  | 1.50 | 0.228 | 1.000 | 0.03 |  | - | - | - |
| LEDD total (mg/day) | 628.92 (263.45) | 469.75 (210.28) | 541.57 (239.35) |  | 2.55 | 0.084 | 1.000 | 0.05 |  | - | - | - |
| LEDD_DA_ (mg/day) | 56.00 (72.45) | 91.73 (129.85) | 49.74 (74.22) |  | 1.44 | 0.241 | 1.000 | 0.03 |  | - | - | - |
| LEDD_L-DOPA_ (mg/day) | 535.42 (274.78) | 319.89 (231.43) | 416.00 (269.65) |  | 3.84 | 0.025 | 0.608 | 0.08 |  | **-** | - | - |
|  |  |  |  |  |  |  |  |  |  |  |  |  |
| *Behavioural measures* |  |  |  |  |  |  |  |  |  |  |  |  |
| Fatigue Severity Scale | 3.05 (2.15) | 2.76 (1.56) | 4.65 (1.55) |  | 11.81 | <0.001 | **<0.001** | 0.22 |  | 1.000 | **0.018** | **<0.001** |
| Beck Depression Inventory | 6.23 (7.03) | 5.88 (5.86) | 13.83 (6.85) |  | 14.54 | <0.001 | **<0.001** | 0.26 |  | 1.000 | **0.003** | **<0.001** |
| Parkinson Anxiety Scale | 10.17 (9.01) | 8.32 (7.75) | 17.34 (9.52) |  | 9.91 | <0.001 | **0.003** | 0.19 |  | 1.000 | 0.050 | **<0.001** |
| Apathy Evaluation Scale | 28.90 (9.36) | 31.75 (7.02) | 32.28 (7.30) |  | 0.79 | 0.457 | 1.000 | 0.01 |  | - | - | - |
| Parkinson’s disease sleep scale | 114.91 (18.67) | 118.68 (23.70) | 110.33 (20.93) |  | 1.06 | 0.351 | 1.000 | 0.02 |  | - | - | - |
| Epworth Sleepiness Scale | 7.29 (5.86) | 4.76 (3.71) | 6.40 (4.14) |  | 2.35 | 0.101 | 1.000 | 0.05 |  | - | - | - |
|  |  |  |  |  |  |  |  |  |  |  |  |  |
| *Cognitive assessment* |  |  |  |  |  |  |  |  |  |  |  |  |
| MoCA total score^b^ | 16.43 (5.10) | 23.71 (2.83) | 23.84 (3.56) |  | 22.97 | <0.001 | **<0.001** | 0.35 |  | **<0.001** | **<0.001** | 1.000 |
| MoCA subscores:^b^ |  |  |  |  |  |  |  |  |  |  |  |  |
| Memory | 0.58 (0.99) | 1.73 (1.60) | 1.14 (1.30) |  | 3.54 | 0.034 | 0.804 | 0.07 |  | - | - | - |
| Visuospatial abilities | 0.49 (1.25) | 0.93 (1.30) | 0.56 (1.51) |  | 0.87 | 0.423 | 1.000 | 0.02 |  | - | - | - |
| Executive functions | 0.47 (2.22) | 1.08 (1.69) | 0.75 (1.61) |  | 0.70 | 0.498 | 1.000 | 0.01 |  | - | - | - |
| Attention, and WM | 4.03 (1.55) | 5.26 (0.79) | 5.22 (0.85) |  | 8.45 | <0.001 | **0.011** | 0.17 |  | **0.001** | **0.001** | 1.000 |
| Language | 2.99 (1.64) | 3.56 (1.19) | 3.20 (1.42) |  | 1.15 | 0.321 | 1.000 | 0.02 |  | - | - | - |
| Orientation | 5.36 (0.74) | 5.95 (0.29) | 5.72 (0.65) |  | 6.63 | 0.002 | 0.051 | 0.14 |  | **-** | - | - |
| PD-CFRS score | 0.83 (0.93) | 0.77 (0.80) | 5.07 (3.44) |  | 38.92 | <0.001 | **<0.001** | 0.48 |  | 1.000 | **<0.001** | **<0.001** |

Note. a, categorical variable; b, adjusted according to age, education, or sex; *ϕc,* Cramér’s V; η^2^, partial eta squared; Adj-*p* represents *p*-value corrected for multiple comparisons using the Bonferroni procedure; SD, Standard Deviation; statistically significant differences are shown in **bold**; UPDRS, Unified Parkinson’s Disease Rating Scale; LEDD, Levodopa Equivalent Daily Dose. O, Overestimators; A, Accurate estimators; U, Underestimators.

**Supplementary Table 4.** Simple and multiple binary logistic regression analyses assessing which demographics, clinical, and behavioural features distinguished Underestimators from Non-underestimators; 95% bias corrected and accelerated confidence intervals [95% CI] (1000 bootstrap samples) for the logistic regression coefficients were reported in parentheses. Analyses were run after excluding five patients with subjective cognitive complaint and objective cognitive impairment.

| Variable | Estimate [CI 95%] | Bias | SE | *p*-value^b^ | OR [CI 95%] |
| --- | --- | --- | --- | --- | --- |
| Simple Regression | |  |  |  |  |
| *Demographics* | |  |  |  |  |
| Age | 0.02 [-0.03, 0.08] | 0.00 | 0.02 | 0.304 | 1.02 [0.97, 1.07] |
| Education, years | -0.11 [-0.23, -0.00] | -0.00 | 0.06 | 0.893 | 0.89 [0.79, 1.00] |
| Sex^a^ | -0.29 [-1.27, 0.72] | 0.00 | 0.48 | 0.526 | 0.74 [0.30, 1.84] |
|  |  |  |  |  |  |
| *Clinical features* |  |  |  |  |  |
| Age at onset | 0.02 [-0.02, 0.08] | 0.00 | 0.02 | 0.273 | 1.02 [0.97, 1.07] |
| Disease duration, years | 0.02 [-0.16, 0.21] | 0.00 | 0.09 | 0.793 | 1.02 [0.87, 1.18] |
| UPDRS-III | 0.01 [-0.03, 0.07] | 0.00 | 0.02 | 0.589 | 1.01 [0.96, 1.06] |
| Hoehn and Yahr stage | 0.77 [-0.27, 2.55] | 0.43 | 2.87 | 0.138 | 2.22 [0.61, 8.07] |
| LEDD total (mg/day) | 0.00 [-0.00, 0.00] | 0.00 | 0.00 | 0.482 | 1.00 [0.99, 1.00] |
| LEDD_DA_ (mg/day) | -0.00 [-0.00, 0.00] | 0.00 | 0.00 | 0.187 | 0.99 [0.99, 1.00] |
| LEDD_L-DOPA_ (mg/day) | 0.00 [-0.00, 0.00] | 0.00 | 0.00 | 0.410 | 1.00 [0.99, 1.00] |
|  |  |  |  |  |  |
| *Behavioural measures* |  |  |  |  |  |
| Fatigue Severity Scale | 0.63 [0.33, 1.09] | 0.03 | 0.18 | **<0.001** | 1.88 [1.37, 2.58] |
| Beck Depression Inventory | 0.17 [0.10, 0.32] | 0.01 | 0.05 | **0.001** | 1.19 [1.09, 1.30] |
| Parkinson Anxiety Scale | 0.10 [0.05, 0.17] | 0.00 | 0.03 | **<0.001** | 1.11 [1.05, 1.17] |
| Apathy Evaluation Scale | 0.09 [0.02, 0.18] | 0.00 | 0.04 | 0.054 | 1.09 [0.99, 1.20] |
| Parkinson’s disease sleep scale | -0.01 [-0.04, 0.00] | -0.00 | 0.01 | 0.179 | 0.98 [0.96, 1.00] |
| Epworth Sleepiness Scale | 0.06 [-0.04, 0.18] | 0.00 | 0.06 | 0.263 | 1.06 [0.95, 1.18] |
|  |  |  |  |  |  |
| Multiple regression^c^ |  |  |  |  |  |
| Fatigue Severity Scale | 0.58 [0.06, 1.52] | 0.07 | 0.39 | **0.011** | 1.79 [1.14, 2.79] |
| Beck Depression Inventory | 0.15 [0.03, 0.46] | 0.02 | 0.10 | **0.019** | 1.16 [1.02, 1.31] |
| Parkinson Anxiety Scale | 0.01 [-0.10, 0.10] | -0.00 | 0.05 | 0.834 | 1.01 [0.93, 1.09] |

Note. a, coded as: 0= male, 1= female; b, *p*-value related to unstandardized beta coefficient by using the Wald statistic; ^c^ Model χ^2^ (3) = 36.86, *p*-value < 0.01, R^2^ = 0.50 (Nagelkerke); SE, Standard Error; OR, Odds Ratio; CI, Confidence Interval; statistically significant variables are shown in **bold**.

**Supplementary Table 5.** Simple and multiple linear regression analyses assessing which demographics, clinical, and behavioural features were associated with the discrepancy between objective and subjective cognitive functioning (MoCA *Z*-scores minus PD-CFRS *Z*-scores) in overall sample; 95% bias corrected and accelerated confidence intervals [95% CI] (1000 bootstrap samples) for the linear regression coefficients were reported in parentheses. Analyses were run after excluding five patients with subjective cognitive complaint and objective cognitive impairment.

| Variable | Estimate [CI 95%] | SE | Bias | β | *p*-value |
| --- | --- | --- | --- | --- | --- |
| Simple Regression | |  |  |  |  |
| *Demographics* | |  |  |  |  |
| Age | 0.02 [-0.03, 0.07] | 0.02 | 0.00 | 0.07 | 0.513 |
| Education, years | -0.10 [-0.21, 0.03] | 0.06 | 0.00 | -0.17 | 0.125 |
| Sex^a^ | -0.15 [-1.28, 0.92] | 0.54 | 0.01 | -0.03 | 0.783 |
|  |  |  |  |  |  |
| *Clinical features* |  |  |  |  |  |
| Age at onset | 0.03 [-0.02, 0.09] | 0.03 | 0.00 | 0.12 | 0.256 |
| Disease duration, years | -0.16 [-0.33, 0.07] | 0.10 | 0.01 | -0.18 | 0.090 |
| UPDRS-III | -0.00 [-0.05, 0.04] | 0.02 | 0.00 | -0.02 | 0.823 |
| Hoehn and Yahr stage | 0.26 [-0.77, 1.28] | 0.53 | -0.01 | 0.03 | 0.723 |
| LEDD total (mg/day) | -0.00 [-0.00, 0.00] | 0.00 | 0.00 | -0.08 | 0.449 |
| LEDD_DA_ (mg/day) | -0.00 [-0.00, 0.00] | 0.00 | 0.00 | -0.08 | 0.496 |
| LEDD_L-DOPA_ (mg/day) | 0.00 [-0.00, 0.00] | 0.00 | 0.00 | -0.06 | 0.598 |
|  |  |  |  |  |  |
| *Behavioural measures* |  |  |  |  |  |
| Fatigue Severity Scale | 0.70 [0.39, 1.02] | 0.16 | 0.00 | 0.51 | **<0.001** |
| Beck Depression Inventory | 0.19 [0.11, 0.26] | 0.04 | -0.00 | 0.55 | **<0.001** |
| Parkinson Anxiety Scale | 0.11 [0.05, 0.16] | 0.02 | 0.00 | 0.42 | **<0.001** |
| Apathy Evaluation Scale | 0.09 [0.02, 0.16] | 0.03 | 0.00 | 0.26 | **0.018** |
| Parkinson’s disease sleep scale | -0.01 [-0.04, 0.00] | 0.01 | 0.00 | -0.13 | 0.267 |
| Epworth Sleepiness Scale | 0.15 [-0.00, 0.33] | 0.08 | -0.00 | 0.25 | **0.022** |
|  |  |  |  |  |  |
| Multiple regression^b^ |  |  |  |  |  |
| Fatigue Severity Scale | 0.39 [0.07, 0.81] | 0.18 | -0.00 | 0.28 | **0.018** |
| Beck Depression Inventory | 0.14 [0.03, 0.26] | 0.05 | 0.00 | 0.42 | **0.003** |
| Parkinson Anxiety Scale | 0.00 [-0.05, 0.06] | 0.03 | -0.00 | 0.03 | 0.806 |
| Apathy Evaluation Scale | -0.01 [-0.07, 0.04] | 0.03 | 0.00 | -0.03 | 0.711 |
| Epworth Sleepiness Scale | -0.00 [-0.12, 0.16] | 0.07 | 0.00 | -0.00 | 0.986 |

Note. a, coded as: 0= male, 1= female; b, Model (F-test) = 10.80, *p*-value < 0.001, R^2^ = 0.41; SE, Standard Error; CI, Confidence Interval; statistically significant variables are shown in **bold**.
